# Entitlement to Sickness Benefits in Sweden: The Social Insurance Officers Experiences

**DOI:** 10.4137/EHI.S858

**Published:** 2008-06-20

**Authors:** Elsy Söderberg, Ulrika Müssener

**Affiliations:** Division of Social Medicine and Public Health Science, Department of Health and Society, Linköping University, S-581 85 Linköping, Sweden. Division of Community Medicine, Department of Medical and Health Science, Linköping University, S-581 83 Linköping, Sweden

**Keywords:** medical consultants, sickness benefit, social insurance officer, assessment, client

## Abstract

**Background:**

Social insurance offices (SIOs) handle a wide range of complex assessments of the entitlement to sickness benefits for an increasing number of clients on sick leave and consequently, the demands on the SIOs have increased considerably.

**Aim:**

To gain deeper knowledge of the problems experienced by the SIOs in their work associated with entitlement to sickness benefits.

**Method:**

A descriptive and explorative qualitative approach was used to analyse data from two focus-group interviews, including six participants in each group.

**Results:**

The participants discussed different dilemmas in regard to; physicians’ responsibility for issuing sickness certificates, interactions with the insured individuals, disclosure of decisions, communications with medical consultants, documentation of sickness benefit claims, threats in the workplace, as well as their own competence. The SIOs regarded incomplete information on sickness certificates as a main problem, because they frequently had to contact the client and the physicians who issued the certificates in order to obtain further details, leading to delays in the decision-making whether to grant sickness benefits.

**Conclusions:**

More knowledge regarding SIOs work is required to improve the methods used in the sickness insurance system and to ensure adequate training of new staff members.

## Introduction

There are two requisites for entitlement to sickness benefits: a person must have a disease or injury and it must be shown that this has caused reduced work capacity. In Sweden, and in most other Western European countries, the physicians are responsible to assess their clients work capacity and to issue sickness certificates, in other words to objectively certify the clients medical situation for other actors, such as the social insurance authority (Alexanderson and Norlund, 2004). The formal decision as to whether a person is entitled to sickness benefit is made by social insurance officers (SIOs) (Hensing et al. 1997). To arrive at decisions, they review information from statements that physicians issue on the certificate and from the sickness benefit recipients themselves.

The tasks of the SIOs have been broadened to include the assessment of measures to facilitate return to work (Edlund, 2001). Besides, they handle a wider range of complex assessments of the entitlement to sickness benefits for an increasing number of clients on sick leave (Hensing et al. 1997), and consequently, the demands on the SIOs have increased (Stendahl, 2003). Their work includes many comprehensive tasks; to control, to co-ordinate and to represent the client. They assess whether self-certifications are complete, determine whether any other benefits restricts the right to sickness benefit, assess the quality of the medical certificates, and make decisions whether to grant sickness benefits. Besides, as mentioned, they evaluate the need for measures to facilitate return to work, or assess whether an application for disability pension should be made. All tasks imply meetings with physicians, employers, handing officers and other professionals involved (Söderberg, 2005b). They dispose a certain degree of decisions latitude, but are at the same time supposed to apply the rules and regulations of the social insurance system. During the last years, the assessment regarding sickness benefits is based on strictly medical basis. For the SIOs, strict applications of rules involves both limitation and protection (Johansson, 1992), which in turn might make it more difficult, but in some cases easier to handle each single case separately. The changes toward assessment based on strictly medial basis are in some cases met with negative criticism among the insured individuals which might be one reason to the fact that SIOs report that they receive various kinds of threats, that has increased in recent years (Söderberg, 2005b). A fundamental dilemma of the work of these actors is that they assess and encounter clients who demands might be in conflict with the policy and resources of the organisation (Söderberg, 2005b; Lipsky, 1984). The core of their work is human contacts, and SIOs are mandated to collaborate also with healthcare professionals and other persons in authority. Similarly, Sawney (Sawney, 2002), indicated that physician’s role as experts in sickness certification is a frequently occurring and stressful task. Research show that physicians in general, perceive these tasks as problematic, and that sickness certificates are often of poor quality (Wahlström and Alexanderson, 2004). Thus, incomplete information on sickness certificates implies that SIOs frequently have to contact the physicians who issued the certificates in order to obtain further details, and delays in decision-making regarding sickness benefits.

There is so far little research on the impact of the practices of SIOs, or their experiences of interaction and cooperation with clients and healthcare professionals (Hensing et al. 1997; Timpka et al. 1995). More knowledge is warranted in this area to facilitate professional development of these welfare professionals, and more attention need to be directed towards the decision making processes.

The aim of the present study was to gain deeper knowledge of the problems experienced by social insurance officers in their work associated with entitlement to sickness benefits.

## The Study

Data from two focus-group interviews; one including six SIOs with short time of employment and on group including six SIOs with long time employment, were analysed.

### Interviewees

Interviewees were strategically chosen from all SIOs working at Försäkringskassan in Linköping and Norrköping, two larger cities in the county of Ö;stergötland. The recruitment was done by their nearest manager. A total of 14 persons were asked to take part in the study, and 12 agreed to participate and were assigned to one of two focus-groups. One group consisted of persons with long period of employment (6 persons). They had all been working on average 20 years, except for one person who had been working for six years at the Försäkringskassan. The other group (6 persons) included those with shorter period of employment. They had all finished university during the last two years and for many, this was their first job. The purpose of this division into two groups was to study whether there were any differences of how they discussed concerning their experiences of their work tasks in regard to periods of employment.

### Focus-group interviews

Focus-group interviews are a well established method of data collection in health research (Morgan and Krueger, 1998; Vaughn et al. 1996). All interviews in the present study were performed in the social insurance office. The moderator explained the purpose of the interview and aspects of confidentiality, and informed the participants that they could withdraw at any time. The interviewees were encouraged to speak freely. Focus of the interview was dilemmas experienced by SIOs in their work associated with entitlement to sickness benefits. An interview guide were developed and used. The task of the moderator was to introduce new topics, to balance the participation of talkative and quiet interviewees, and to continually summarize what was said during the interviews. An observer, sitting outside the group had the task of controlling that all questions were discussed about. The focus-group interviews lasted approximately one and a half to two hours.

### Data analysis

The focus-group interviews were audio taped and transcribed verbatim. Analyses of the data has been performed by using an descriptive and explorative qualitative approach (May, 1997). The analyses were performed at the group level, and little effort was made to identify individuals who made certain statements or to note the frequency or intensity of certain comments (Krueger and King, 1998). One of the author (ES), and another researcher individually read all interviews several times (Krueger and King, 1998). Each person independently identified statements about SIOs experiences of dilemmas in their work. The chosen quotations were then compared and discussed until agreement was reach on which statements to include. Pronouncements that were not agreed on to be clearly indicating dilemmas were excluded. Thereafter patterns were search for in the identified quotations, six categories were formed, and boundaries for the categories were established. During this process several discussions were held. The quotations were scrutinised by the other author (UE). Statements not agreed on were excluded. Excerpts from the interview transcripts are presented below to support and illustrate the categorisation.

## Findings

In the presentation of results, the terms insured individual and client are used as synonyms. The term medical consultants refer to physicians working at the social insurance office and the term physicians refer to physicians who are responsibility for issuing sickness certificates. This section summarises the way the SIOs in the two focus-groups described their contacts with the healthcare and medical services. Above all, their discussions concerned physicians responsible for sickness certification, interactions with the insured individuals, disclosure of decisions, communications with medical consultants, documentation of sickness benefit claims, and threats in the workplace, as well as issues concerning competence and other views on the sickness certification process.

### Contacts with healthcare and medical services

The SIOs regarded incomplete information on sickness certificates as a major problem, because they frequently had to contact the physicians who issued the certificates in order to obtain further details. Both of the focus groups discussed whether incomplete sickness certificates should be sent back to the insured individuals, who in turn would be responsible for contacting the issuing physicians. The point would be to show the physicians how important it is to provide sufficient information from the start. The alternative mentioned indicated that in some cases the SIOs should get information directly from insured individual.

Members of the two focus groups described their contacts with the certifying physician in somewhat different ways. Most of the study participants agreed that their contact with medical consultants was good, and they considered those physicians to be particularly important when initiating a more detailed assessment of a claim. Nevertheless, the SIOs found it difficult to explain to their clients what role the medical consultants play in social insurance cases. In general, the SIOs with longer periods of employment thought that the communication with the certifying physicians had improved, although they said that some physicians took offence when asked to provide information that was missing on sickness certificates. These participants also felt that the high turnover of physicians at healthcare centres and hospitals posed a problem. Handling of a case can be delayed if it is necessary to acquire supplementary information from a physician other than the one who originally issues a certificate, which in turn will have a negative impact on the insured individuals. On the whole, the SIOs indicated that it would be a good idea for physicians to inform clients about the different roles played by the healthcare and medical services and the SIOs in the sickness certification process.

Some of the SIOs with short periods of employment mentioned that contact with the physicians responsible for sickness certification is inadequate. In their opinion, this is partly due to the physicians meaning that the information they provide on the certificates is doubted by SIO staff, and that they sometimes regard the SIOs as opponents rather than associates in the process of helping their insured individuals obtain sickness benefit when there is medical causes for the granting of sickness benefits. These SIOs found it difficult to understand why physicians could not provide better the required information in the certificates, as suggested by the following:

“I think it’s hard to understand why the physicians can’t cooperate by issuing better sickness certificates that they don’t make an effort to write legibly. And they misinterpret us sometimes when we ask for additional information. It’s not our intention to question their competence, but rather to obtain information that they didn’t provide [in the first place]. Our collaboration should be facilitated; they should listen to us instead.”

Both groups of respondents talked about instances when physicians in charge of sickness certification were requested to provide supplementary information to aid decisions about sickness benefit payments, and it became apparent that no further information was available on a client who had previously been issued a sickness certificate.

The SIOs said: “Sometimes there is no more information, which means that a sickness certificate was issued even though the client wasn’t sick, and that’s odd.”

It was also apparent that the SIOs felt that the quality of certificates had improved.

All of the participants indicated that it is difficult for both physicians and their clients to understand that those who are unemployed must consider all parts of the labour market when looking for jobs.

“A client looks for available jobs as a carpenter and then we say ‘but you could try to find some lighter work since we’re considering the entire labour market.’ But there are even physicians who think it’s difficult to understand why [their clients] are trying to find work as carpenters.”

A problem that was highlighted in the discussions was that the physicians who handle sickness certification often know very little about their clients’ employment status, occupation or how assessment of unemployment is done. This is illustrated by the observation that the certifying physicians find it more difficult to assess disease or injury in relation to reduced work capacity than to validate the existence of disease or injury according to the rules and regulations of the sickness insurance.

### Contact with insured individuals

The participants in the focus groups talked about different kinds of interactions with their clients. One topic dealt with the questions that clients ask when they are not granted the sickness benefit they feel they are entitled to and contact the SIO because they are waiting for the decision on the granting of sickness benefits. The SIOs meant that such conversations are trying, partly because they are time consuming, but also because it is often difficult to provide the exact information that the clients want.

The SIOs also discussed examples of when they themselves contacted their clients to obtain supplementary information, and they regarded such communication as a significant part of their work. The discussions also indicated that the purpose of case management is to discern other signals of activity related to sickness certification, and emphasised that the social insurance office does not want clients to get stuck in the sick role. In this context, it was also mentioned that contact with employers is helpful.

“But I don’t want them to just accept being on sick leave, that’s when it’s a good idea to focus on [the issue], if a return to work meeting has been scheduled. Then I think it’s really smart to phone the employer and ask when the meeting is, as well as some other things.”

The SIOs were under the impression that most people on sick leave assume that a sickness certificate automatically entitles them to sickness benefit. However, that document must confirm that there is a disease, illness or injury to entitle the client’s right to such compensation, and the SIOs found it is easier to explain this orally than in a letter. Members of both focus groups felt that the most difficult thing about the initial assessment of work capacity is that there is insufficient information in the sickness certificates. The study participants also discussed the clients’ responsibility to submit documents, and they felt that they talked to their clients on the phone more often today than in the past.

“Anyway, it’s our task to steer the conversation, and sometimes you feel like you’re losing control, and it’s the client who’s in command. It doesn’t matter what you ask, because they’re doing the talking. I feel like I’m in control when I’m asking questions. It doesn’t always work, sometimes you feel like you just have to let it go.”

Both groups indicated that a more rigid assessment of a case entails a much larger number of contacts with the client, and those dialogues frequently involve explaining the changes that have recently been made in the social insurance system; that sickness benefits should be granted based on strictly medical grounds:

“Then I think that the client can give us the documents that it’s up to the [benefit] applicant to submit the documents so that we can make a decision. One client asked me: ‘Why didn’t you get more information from my physician? We did, but you’re the one who is applying for benefit payment and wants compensation, which means it’s your responsibility to give us the [necessary] documents.’ It’s difficult, because it didn’t used to be like that.”

The SIOs with shorter period of employment talked extensively about how they conducted discussions and meetings with clients in a professional manner. They felt that the responses from their clients were very positive, although many conversations did focus on frustration and disappointment in relation to both the social insurance office and society in general.

Knowing when to terminate an unproductive dialogue with a client was regarded as problematic. The members of the other focus group said that it is more difficult to maintain distance and integrity when handling clients who are unhappy, since such individuals tend to affect the SIOs more personally, making it harder for them to “disconnect themselves” because they feel emotionally involved.

### Threats in the workplace

In the focus groups, the SIOs indicated that they are confronted with several types of threats, some directed towards them in their official work, and others involving clients that threaten themselves, for instance, harming themselves. The focus group members found it difficult to know how to handle such situations in the “right” way, and they doubted or were not sure that they make or did correct judgements and decisions under those circumstances.

The participants agreed that the number of clients that threaten to harm themselves has increased. Early in the focus group discussion, the SIOs with shorter period of employment broached the subject of clients threatening to harm themselves. One participant had felt uneasy about going home from work during the period of handling a very demanding case. The names of social insurance staff involved are included in the claim documents, and hence SIOs can feel vulnerable. Members of the focus group comprising long-service employees also said that the number of threat situations had increased. They suggested that this might be explained by the fact that applications for sickness benefit payments are being rejected more extensively now than was previously the case, and that that could make clients angry and desperate. Furthermore, they indicated that it is difficult to know how to handle such situations in the right way—they felt professionally inadequate. They also said that threats from clients are very taxing, but support from helpful colleagues makes it easier to accept what has happened. Being sensitive to each other’s concerns when such situations arise was regarded as extremely important. One of the participants said the following:

“I’ve felt threatened sometimes but luckily not very often, although it feels kind of like more things are about to happen now compared to the way it used to be.”

All of the study participants had at some time felt anxious about unscheduled visits from clients, especially when such meeting were preceded by an unpleasant phone call with the client in question. It can feel highly disagreeable to confront a client face-to-face under such circumstances. One of the participants described the need for more protection when greeting clients in the reception area. This was compared with other professions that entail contacts between authorities and members of the general public, and that have a different type of organisation for receiving clients that might behave in a threatening manner.

### Difficulties in making decisions

The discussions indicated that SIOs find it difficult to evaluate illnesses and diagnoses. Considering sickness certification, this is reflected by an increasing number of clients seeking treatment for symptoms or complaints for which there is no standardised method for assessment of a specific medical condition, and hence physicians give vague descriptions on the certificates.

Apparently, this is more common among people with different types of fatigue and pain conditions. All of the focus group participants agreed that it is difficult to determine the right to sickness benefit based on symptoms that are summarised on sickness certificates as exhaustion, anxiety, and crisis reaction. Such certificates usually give no information about what the consequences of the medical condition in question will have for the client’s capacity to work. The SIOs also indicated a pronounced increase in psychiatric diagnoses, symptoms of mental illness, exhaustion, and subjective experience of ill health:

“And in those cases you often get unclear information from the sickness certificates. What do things like crisis reaction, exhaustion, and despondency mean? Exhausted and despondent, I can feel like that and go to work anyway, and still figure out how serious a case is and why that particular client can’t work. How am I supposed to determine whether a client can work half time or full time, when [the physicians] describe common or general things instead of stating that this client can’t manage his or her job due to this or that.”

The SIOs with long period of employment also deliberated about what diagnoses such as crisis reaction actually mean. They discussed problems related to determining whether a crisis reaction is serious enough for the client to require six weeks to recuperate, or if the condition is less severe so that the client can already start to work part time. The participants strongly agreed that it is difficult to make a prognosis regarding return to work for clients who suffer from such medical conditions. One of the SIOs stated the following about information concerning the effects of an illness on functional capacity:

“They don’t write that it’s serious. Sure, you understand if it says that they have severe anxiety and all that, but if it says they’re tired or anxious. I can feel like that even though I go to work.”

There was also discussion about how difficult it is to determine whether illness is the reason for reduced work capacity in pregnant women. Assessments of such clients represented issues that the SIOs can relate to their own experiences and/or to similar problems that had confronted close relatives, which might make it more difficult to reach decisions:

“Pregnant women and problems normally related to pregnancy, in such cases there is no confirmed illness, and applications for sickness benefit are denied of course, because there is no illness, and we have lots of cases like that and they’re hard to handle.”

Some of the SIOs with shorter period of service at the social insurance office brought up the subject of how difficult it is to explain the system of social insurance rules in a didactic manner. In conjunction with that, they emphasised the importance of conveying decisions in an effective and correct way. For example, they felt that it is much more difficult to handle clients that have a different ethnic background and do not speak Swedish as their native language. They also encountered problems in knowing how many times they should request further information from a client before communicating a decision, such as rejection of a claim for sickness benefit.

For certain diagnoses, the SIOs could accept that a client originally had the right to sickness benefit, but that it would be necessary to obtain clearer medical confirmation on a subsequent occasion. They indicated that communication with the insured client made them better prepared to understand the decision that was to be made:

“Then the client is better prepared for us making more extensive demands later on in the case, so that even if we did grant payment from the beginning, the insured client is aware that the groundwork will have to be more rigorous next time.”

Uniformity in evaluations was discussed and considered to be important. Since all clients are different, the study participants were uncertain whether or not they should allow special circumstances to influence their cases:

“But you know it’s pretty important because there are still lots of assessments, and you and I should judge such and such the same, so that it doesn’t turn out that just because a client ends up on my desk he gets sickness benefit payment, whereas he wouldn’t have if he’d ended up on your desk instead.”

The participants with shorter period of employment also mentioned that they did not always have sufficient knowledge to answer queries from clients about what the social insurance office could “provide.” Furthermore, they experienced problems related to not having the authority to give general advice about where their clients can obtain such information.

All of the study participants agreed that their contact with medical consultants was supportive when performing assessments of certificated with unclear information, and they considered those physicians to be particularly important when initiating a more detailed assessment of a claim. Nevertheless, the SIOs found it difficult to explain to their clients what role the medical consultants play in social insurance cases. After having consulted and been treated by their own physicians, insurance applicants were disinclined to accept that those physicians could regard them as being ill, whereas an insurance physician that they had never even met judged that their medical conditions or symptoms did not reduce their capacity to work to such an extent as to entitle them to sickness benefit payments. There was even some uncertainty about when SIOs should contact medical consultants to obtain additional information regarding sickness certification. This problem usually arises due to incomplete sick and the need for help with interpreting the information provided on those documents.

### Opinions about the case management process

The opinions of the study participants differed with regard to documentation of cases, although all of them felt the need for guidelines concerning what should be reported. The SIOs with short period of employment did not have any problems finding appropriate ways to present information in the documents, whereas those with longer service found that task more complicated and emphasised that they often had to write the exact same thing several times and that it was difficult to decide what information should be included. They had received feedback indicating that they include too many details in the records, and not always the information that is really needed. One comment about documentation indicated that the results vary even if they are intended to be the same:

“And then everyone has done it differently, and all the employees there have different personalities, which is noticeable in the way they keep records. Some write a lot and some only a little, some give detailed information and some don’t write anything at all. Then it’s not consistent. We don’t have any real guidelines either, we write the way we want to, without any main points. But it’s going to get better.”

Some of the SIOs with shorter service said that it is hard to see the results of their work, since they do not get any feedback about what they do, nor are they informed of the outcomes. They only become aware of the results if clients call to ask about progress in their cases. They also mentioned that it is difficult to know what colleagues who take over their cases think about what has already been done, largely because individual SIOs have very clearly defined tasks. The division of tasks was described as follows:

“We’re so divided up that it’s like a factory, sort of just get on with the next one.”

Among the SIOs with longer periods of service, there was extensive discussion about the possibility of being able to deal with a case without being interrupted by telephone calls from clients. They also mentioned that they frequently had to talk on the phone to provide information that in their opinion could be provided by the social insurance staff. One of the participants stated the following:

“If you have a phone, I think you should sit by it, because it takes so much energy and there are interruptions because we’re so involved in our work and keep records and write even more about everything. There are so many things you have to remember and that you easily lose track of when you have to answer the phone, and then you forget and then the case is over for your part because someone else has taken it over, and then it doesn’t feel right to double check.”

### Maintaining and developing competence

All of the participants felt that they have multifaceted work tasks that require considerable proficiency and professional performance with respect to knowledge about social insurance, treatment of clients, and communication skills.

The SIOs with less work experience pondered the fact that many people both within and outside the organisation question the need for a college education to handle work at the social insurance office. They considered that opinion odd, because their tasks entail being able to scrutinise data, handle documentation, formulate prognoses, and make correct decisions while working against the clock. They mentioned scepticism towards university graduates working at the social insurance office, as exemplified by this comment:

“It’s rather condescending. We have to start by saying we’re proud. You have to fight to make things right. It’s been given a negative label. That’s strange.”

Several of the participants with short service regarded the cases that were a little more complicated as the most interesting. In that context, they said that interactions with the medical consultants-gave them the opportunity to reflect and learn.

The SIOs with long period of employment discussed their work tasks partly from the perspective of previous experience, but also in relation to how their earlier situation compared with their present circumstances at work. Some pointed out that it is important that a case manager who takes over a claim from a colleague does not overzealously examine or question the previous assessments in front of the client.

The participants were satisfied with the social insurance office work routines that were created to meet the requirements of the new “more rigorous” form of assessment. They also felt that they continuously improved their knowledge and skills through participation in complex evaluations and interactions with physicians. Members of both focus groups agreed that it had been easy to adopt stricter application of the legislation. The SIOs with long service agreed with that, as shown by the following comment:

“We don’t want to go back to the way we used to do it, since it’s actually the way we do the job now that makes it meaningful. That even applies to the clients, because if we don’t stick to it, the assessments will be different.”

Notably, the SIOs with short employment found it surprising that their organisation does not cooperate with other authorities, such as the local tax offices, since the information they compile can be of mutual interest to the respective agencies.

## Discussion

The aim of the present study was to gain deeper knowledge of the problems experienced by social insurance officers in their granting of sickness benefits.

The experiences of the SIOs in their daily work could be divided into six categories referred to as:

contacts with healthcare and medical services,
contacts with the insured individual,
threats in the workplace,
difficulties in making decisions,
opinions about case management process and
maintaining and developing competence.

### 

#### Methodological considerations

An explorative approach was used to identify and analyse how SIOs experiences the process concerning entitlement to sickness benefits. It should be noted that our data are based on a relatively small sample, and the results cannot be generalized to other groups. Nonetheless, we contend that it has provided sufficiently interesting results to motivate further investigations concerning the problems experienced by the SIOs in their work associated with entitlement to sickness benefits

The focus-groups interviews is unique from for example individual face-to-face interviews and questionnaires; it allows for group interaction and greater insight into why certain opinions are held (Kreuger, 1993). The participants often share their ideas and perceptions, and the members influence each other by responding to ideas and comments in the discussion. Focus groups are composed of people who are similar to each other. The nature of this homogeneity is determined by the purpose of the study and is a basis for recruitment (Kreuger, 1993). In the present study, the division into groups was based upon these guiding principles. However, people who regularly interact, for example colleagues, might present special difficulties for the focus-group discussion because they may be responding more on past experiences, events, or discussions than on the topic of concern. But, since emotional aspects of interaction can be a sensitive issue to discuss for a group who have not previously met (Morgan and Krueger, 1998), and such discussion occurred spontaneously in both groups, the group cohesiveness proved to be successful for the purpose of the study. The benefit of homogeneous in the present study is that participants recognized each other’s experiences and could associate them with similar perceptions, which also led to a willingness to share personal experiences.

Validity in qualitative studies is closely associated with the choice of design and with the method used to collect data (Patton, 1990). The validity, meaningfulness, and insights generated by qualitative inquiry have more to do with the richness of the information held by the people being interviewed and the analytical capabilities of the researcher, than with the sample size. Therefore, it is difficult to choose the number of interviewees. That selection can be guided by time and resources, together with the quality of the information received (Patton, 1990). The data obtained in the present interviews were of good quality and gave a broad and distinct picture of the situations of the interviewees. Several steps were taken to ensure the validity of the results (Krueger and King, 1998). The interviewer was a trained group leader and had experience of working with individuals on sick leave, and had also conducted field investigations of clients receiving healthcare. Three persons read the interview transcripts independently many times. Quotations were first selected separately, and then compared and discussed. Statements not agreed on by the authors and the other researcher was excluded.

#### Discussion of results

A review of the literature on practices of SIOs (Söderberg, 2005a), revealed that very few studies have investigated the process of granting sickness benefits, while most of them have focused on cooperation between actors and on the return to work process. Hensing et al. (Hensing et al. 1997) has elucidated the problems experienced by the SIOs in their work associated with entitlement to sickness benefits. SIOs experiences of assessing applications for disability pensions after the government’s introduction of stricter regulations has also been studied (Ydreborg et al. 2007). From a general point of view, our findings concerning dilemmas in SIOs work are in accordance with the results of previous research.

The essence of the discussions in the present study was difficulties in making decisions, and SIOs bring out a strong intention of conveying decisions in an effective and correct way. Incomplete information on sickness certificates was mentioned as the main reason for delays in decisions and contacts with physicians were described as complicated. The discussions indicated that SIOs in general find it difficult to evaluate and assess illnesses and diagnoses and that the certificates usually give no information about what the consequences of the medical condition in question will have for the client’s capacity to work. The lack of standardised methods for assessment was emphasized. Uniformity in evaluations, whether or not one should allow special circumstances to influence decision-making was a central theme, leading to the question whether to use strict or flexible applications of rules. Previous studies in the area has highlightened the fact that the assessment of work capacity represents both important and complex tasks that SIOs must perform without having access to either scientific knowledge or consensus agreement on which to base their decisions (Söderberg et al. 2008). It is of importance to gain knowledge on how to create, develop, and maintain the cooperative competence among SIOs. Studies have shown that sickness certificates have a substantial impact on SIOs judgements regarding the right to sickness benefits (Hensing et al. 1997; Söderberg, 2005a), and consequently, it is of great importance that the certificates are of good quality to facilitate decision making. Various times, the SIOs discussed whether incomplete sickness certificate should be sent back to the clients, who in turn would be responsible for contacting the physician, in order to show how important it is to provide sufficient information from the start. They also mentioned difficulties in knowing how many times they should request further information from a client before communicating a decision, such as rejection of a claim for sickness benefit. Receiving the medical certificate in reasonable time has also been reported as problematic by SIOs in a study by Ydreborg et al. who in accordance with the present study reported incomplete certificates as time-consuming. (Ydreborg et al. 2007).

As a consequence of the incomplete information on the sickness certificates and a more rigid assessments based on strictly medical grounds, the SIOs claimed they have a much larger number of contacts with the clients and the physicians. The dialogues with the client frequently involve explaining the changes that have recently been made in the social insurance system, and were experienced as very time-consuming. They also stressed how difficult it is to explain the system of rules in a didactic manner. In general, the responses from their clients were very positive, although many conversations did focus on irritation and disappointment in relation to both the social insurance office and society in general. In both focus-groups SIOs stressed that it would be a good idea that physician should inform clients about the different roles played by the healthcare and medical services and the SIOs in the sickness certification process. In Sweden and many other industrial nations, the county councils or corresponding organisations (with which physicians are affiliated) and the sickness insurance system (where the SIOs work) have been developed without any cooperation (Lindqvist, 2000). Studies regarding the interaction between the certifying physicians and the SIOs with regard to the way they communicate with each other, or how they reach a common understanding of their tasks, are lacking (Söderberg, 2005b).

Making decisions concerning entitlement to sickness benefits can be described as a process of learning and becoming proficient at the tasks to be performed (Jönsson et al. 2004). Lassbo (Lassbo, 2003) claim that an occupational group who strive to be considered as professional must receive legitimacy within the environment they work in. Professionalism is a matter of the relation between the professionals, their clients and the society. A member within a professional organization must be able to show that he or she possesses practical experience concerning the current problem. The client, in this case the sick listed client, must accept this knowledge and be willing to profit by it. It is the professional skill that is the crucial determining factor whether the group reaches acknowledgement or not (Lassbo, 2003).

Talking about competence, the results of the present study indicated that SIOs with less work experience pondered the fact that people both within and outside the organisation questioned the need for a college education to handle their work tasks. They considered the opinion odd, since their tasks entail being able to assess information and handle such data, formulate prognoses, and make decisions while working against the clock. Further, they were frustrated over the lack of uniform and explicit directives about applicable routines on the whole. Previous research regarding the role of these administrators present that SIOs as a profession do not represent a homogeneous group and the members seem to have different backgrounds and education. A majority of them have long work experience and some have gained competence through higher education and/or types of work other than social insurance administration. That SIOs have different backgrounds can lead to a weak profession identity (Edlund, 2001; Hall, 2001). Lack of a distinct formulated professional role leads to problems, which can be related to the organisation, but instead can be attributed to a lack of competence in SIOs (Söderberg, 2005b). In general, a explicit professional identity might facilitate for employees to respond to and cooperate with other actors in a professional manner (Pingel and Robertson, 1998). This might be the case also for SIOs.

Despite the dilemmas reported regarding entitlement to sickness benefits, the SIOs were however satisfied with the social insurance office work routines that were meant to meet the requirements of the new more rigorous form of assessment. Members of both focus groups agreed that it had been relatively easy to adopt stricter application of the legislation, and that contact with physicians and the insured individuals in general has improved. However, SIOs also indicated that the changes toward assessment based on strictly medial basis has resulted in an increasing number of threats, both directed towards them and other involving clients that threaten themselves. The participants stressed that it is difficult to know how to handle such situations in the right way and that they felt professionally inadequate. As work-place violence gains increasing recognition as an issue of major concern (Arnetz, 2000), there is a growing need for research that focuses on preventive and intervention strategies. By striving for college education, more distinct formulated professional roles, complete information on sickness certificates, and greater cooperation with physicians, the number of threats might decrease.

## Implications for Practice

Knowledge regarding dilemmas in SIOs work is required to improve the methods used in the sickness insurance system and to ensure adequate training of new staff members. SIOs need to develop their awareness of, and skills in, stimulating the insured individuals to develop their own plans to improve their situation. A clinical implication elucidated is the need for professionals to be conscious of, and further develop, their cooperation with other practitioners involved in the process associated with the entitlement to sickness benefits.

## Conclusions

The lack of knowledge concerning the practices of SIOs is remarkable given the substantial economic burden on society that exists due to administration and payment of sickness compensation. SIOs have multifaceted work tasks that require considerable proficiency and professional performance with respect to knowledge about social insurance, treatment of clients and communication skills. Further studies need to examine cooperation with other professionals involved in the administration of sickness insurance, for instance, insurance physicians. The conceptual and theoretical framework in this area needs to be developed to facilitate elucidation of the interaction between different actors in local spheres, between different professionals, and between welfare staff and insured individuals.
